# Risk prediction model for cardiovascular diseases in adults initiating pharmacological treatment for attention-deficit/hyperactivity disorder

**DOI:** 10.1136/ebmental-2022-300492

**Published:** 2022-09-05

**Authors:** Maja Dobrosavljevic, Seena Fazel, Ebba Du Rietz, Lin Li, Le Zhang, Zheng Chang, Tomas Jernberg, Stephen V Faraone, Johan Jendle, Qi Chen, Isabell Brikell, Henrik Larsson

**Affiliations:** 1 School of Medical Sciences, Örebro University, Örebro, Sweden; 2 Department of Psychiatry, University of Oxford, Oxford, UK; 3 Department of Medical Epidemiology and Biostatistics, Karolinska Institutet, Stockholm, Sweden; 4 Department of Clinical Sciences, Danderyd University Hospital, Stockholm, Sweden; 5 Departments of Psychiatry and of Neuroscience and Physiology, SUNY Upstate Medical University, Syracuse, New York, USA

**Keywords:** Adult psychiatry

## Abstract

**Background:**

Available prediction models of cardiovascular diseases (CVDs) may not accurately predict outcomes among individuals initiating pharmacological treatment for attention-deficit/hyperactivity disorder (ADHD).

**Objective:**

To improve the predictive accuracy of traditional CVD risk factors for adults initiating pharmacological treatment of ADHD, by considering novel CVD risk factors associated with ADHD (comorbid psychiatric disorders, sociodemographic factors and psychotropic medication).

**Methods:**

The cohort composed of 24 186 adults residing in Sweden without previous CVDs, born between 1932 and 1990, who started pharmacological treatment of ADHD between 2008 and 2011, and were followed for up to 2 years. CVDs were identified using diagnoses according to the International Classification of Diseases, and dispended medication prescriptions from Swedish national registers. Cox proportional hazards regression was employed to derive the prediction model.

**Findings:**

The developed model included eight traditional and four novel CVD risk factors. The model showed acceptable overall discrimination (C index=0.72, 95% CI 0.70 to 0.74) and calibration (Brier score=0.008). The Integrated Discrimination Improvement index showed a significant improvement after adding novel risk factors (0.003 (95% CI 0.001 to 0.007), p<0.001).

**Conclusions:**

The inclusion of the novel CVD risk factors may provide a better prediction of CVDs in this population compared with traditional CVD predictors only, when the model is used with a continuous risk score. External validation studies and studies assessing clinical impact of the model are warranted.

**Clinical implications:**

Individuals initiating pharmacological treatment of ADHD at higher risk of developing CVDs should be more closely monitored.

**WHAT IS ALREADY KNOWN ON THIS TOPIC**

- Emerging research indicates that adults with attention-deficit/hyperactivity disorder (ADHD) are at increased risk for several traditional cardiovascular disease (CVD) risk factors, such as hypertension, obesity, smoking, type 2 diabetes and hyperlipidaemia.
- Prediction models have been developed to identify individuals at high-risk of CVD, yet they have not been validated in adults with ADHD, for whom they may not accurately predict the risk.

**WHAT THIS STUDY ADDS**

- The current study considered novel CVD risk factors which are associated with ADHD (ie, comorbid psychiatric disorders, sociodemographic factors and other psychotropic medication).
- Novel risk factors may improve the prediction of CVDs in adults initiating ADHD pharmacological treatment compared with a model with traditional CVD predictors only.

HOW THIS STUDY MIGHT AFFECT RESEARCH, PRACTICE OR POLICY

- A CVD prediction model optimised to individuals with ADHD could assist clinicians to identify high-risk individuals at ADHD pharmacological treatment initiation.

## Background

Cardiovascular diseases (CVDs) are the leading cause of death and disease burden among adults globally.^1^ Emerging research indicates that adults with attention-deficit/hyperactivity disorder (ADHD) are at increased risk for several traditional CVD risk factors (eg, hypertension, obesity, smoking, type 2 diabetes and hyperlipidaemia).^2 3^ Additionally, the stimulant (ie, amphetamine-based and methylphenidate-based medications) and non-stimulant (ie, atomoxetine) medications for ADHD increase blood pressure and heart rate by increasing the activity of the sympathetic nervous system,^4^ although serious cardiovascular events (ie, stroke, ischaemic heart diseases) are rare.^5^

To optimise preventative strategies for CVDs, prediction tools have been developed to identify individuals at high risk of CVD. Several well-established risk prediction models for CVDs have been validated in different populations and implemented in clinical practice (eg, the Framingham Risk Score).^6^ Yet, such models have not been validated in adults with ADHD, for whom they may not accurately predict the risk. Existing CVD risk prediction models have mostly overlooked novel CVD risk factors such as other psychiatric disorders (ie, anxiety, depression, bipolar disorder, schizophrenia, sleep disorders, substance use disorder), adverse socioeconomic factors (ie, low educational attainment), and the use of other psychotropic medication,^7–12^ variables which are also associated with ADHD.^13 14^ Several professional societies have issued guidelines regarding initiation of pharmacological treatment of ADHD in adults in relation to CVD risk. The National Institute for Health and Care Excellence (NICE) recommends general physical health assessment and an ECG in persons with coexisting cardiovascular issues or conditions treated with medications posing an increased risk for cardiovascular disorders.^15^ The Updated European Consensus Statement on diagnosis and treatment of adult ADHD recommends an assessment of heart rate, blood pressure and weight before treatment, yearly monitoring of these measures, and routine echocardiograms in adults older than 50 years of age.^16^ A CVD prediction model optimised to individuals with ADHD, which would include relevant novel risk factors, could potentially assist clinicians to identify high-risk individuals by providing higher accuracy compared with considering traditional risk factors only. Individuals at high CVD risk could be monitored more often and recommended an individualised treatment plan. Prediction models considering rare, but relevant adverse, outcomes in different psychiatric populations have already been developed and can be applied by clinicians via Web calculators (ie, suicide risk in severe mental health illness^17^ or cardiometabolic risk in young people with psychosis.^18^

## Objective

In the current study, we aimed to develop a 2-year risk prediction model of CVDs in adults initiating ADHD pharmacological treatment using largescale data from population-based health registers from Sweden. We aimed to evaluate the extent to which the predictive accuracy of a model including traditional cardiovascular risk factors (ie, age, sex, hypertension, diabetes mellitus, obesity, hyperlipidaemia and tobacco use disorder)^19 20^ could be improved by adding novel CVD risk factors associated with ADHD (ie, comorbid psychiatric disorders, sociodemographic factors and other psychotropic medication).

## Methods

The study is reported in accordance with the Transparent Reporting of a multivariable prediction model for Individual Prognosis or Diagnosis (TRIPOD) guidelines.^21^ Study protocol is provided in online supplemental appendix H.

10.1136/ebmental-2022-300492.supp1 Supplementary data

### Data sources and study cohort

Data from multiple Swedish national registers were merged using the unique personal identity number assigned to all persons registered in Sweden.^22^ We extracted information on date of birth, sex, date of death, date of migration and country of birth from the Total Population Register, which includes all individuals born since 1932, and who were alive in 1963 onwards.^22^ Information on relevant diagnoses was obtained from the National Patient Register (NPR) and the Cause of Death Register (CDR) and classified according to the International Classification of Diseases (ICD) versions 7/8/9/10. The NPR covers all primary and up to eight secondary diagnoses from inpatient care since 1987, and outpatient care since 2001,^23^ and the CDR contains information on cause of death for all deaths since 1952.^24^ The Prescribed Drug Register (PDR) covers all dispensed medications since 1 July 2005, coded in accordance with the Anatomical Therapeutic Classification (ATC) system. Educational attainment was obtained from Longitudinal integration database for health insurance and labour market studies register^25^ and Census.^26^ All individuals in the study population were linked with their first-degree relatives (parents and full siblings) using the Multi-Generation Register^27^ and information on family history of CVDs was extracted from the NPR.

### Population and study period

Our cohort consisted of individuals born between 1932 and 1990, who started pharmacological treatment of ADHD^28^ between 1 January 2008 and 31 December 2011, at age≥18 years, and who were without previously registered diagnosis of CVDs. The inclusion period started on 1 January 2008, to allow for a wash-out period of 2 years for a previous dispensation of medication for ADHD (ie, a 2-year period free of medication prescriptions for ADHD). The inclusion period ended on 31 December 2011, to allow for at least 2 years of follow-up, since we had access to the data in the registers until 31 December 2013. Individuals were followed from the first ADHD medication dispensation until the date of CVD diagnosis/medication dispensation, emigration from Sweden, death or by the end of 2 years, whichever occurred first.

We included individuals prescribed with stimulant medications: amphetamine (ATC code N06BA01), dexamphetamine (ATC code N06BA02) and methylphenidate (ATC code N06BA04), and non-stimulant medication: Atomoxetine (ATC code N06BA09). Prescriptions that were returned to pharmacies by patients after dispensation and prescriptions for indications other than ADHD (eg, narcolepsy, multiple sclerosis, idiopathic hypersomnia, pain, handicap and catalepsy) were previously removed using natural language processing models for free-text prescriptions from the PDR.^29^

### Predictors

We selected candidate predictors based on previously established CVD risk prediction models and relevant literature (ie, systematic reviews and meta-analyses when available) and expert opinion (see online supplemental appendix A, for the full list of included candidate predictors). Traditional CVD risk factors^6^ available in Swedish national registers, are the following: age at treatment start, sex, a diagnosis or dispensed medication prescription for hypertension, diabetes mellitus (type 1 and type 2 diabetes) and hyperlipidaemia; a diagnosis of obesity and tobacco use disorder; and family history of CVD (first degree relative diagnosed with CVD before age 60).^30^ ^s31^

We considered the following novel CVD risk factors,^7–12^ which have also been shown to be associated with ADHD^13 14^: anxiety, depression, bipolar disorder, schizophrenia, alcohol use disorder, substance use disorder other than tobacco and alcohol, previous use of other psychotropic medications (ie, anxiolytics, antidepressants, hypnotics and sedatives, antiepileptics, mood stabilisers, antipsychotics and drugs used for addictive disorders) and sociodemographic factors: low educational attainment and country of birth (Sweden and other than Sweden). ICD and ATC codes for diagnoses and medication prescriptions for both traditional and novel risk factors were extracted from the NPR and PDR (online supplemental appendix B). Extracted diagnoses, medication prescriptions and sociodemographic information were present by the date of ADHD pharmacological treatment initiation. The current model did not test for interactions between risk factors as there was no theoretical basis for this.

We used a limited backward stepwise procedure to determine whether to retain novel predictors in the model based on their p values, as it has been done previously.^17^ Novel risk factors with the highest p value were sequentially rejected, until none of them remained with a p-value greater than 0.1, while holding fixed traditional risk factors (see Process of predictors selection, online supplemental appendix D).

### Outcomes

CVDs were defined as the first diagnosis (ie, primary or any secondary diagnosis) or dispensed medication prescription for any of the following CVDs: ischaemic heart disease, cerebrovascular disease, venous thromboembolism, heart failure and tachyarrhythmias during the follow-up. CVDs were defined based on ICD-10 codes from the NPR and CDR, and dispensed medication prescriptions for CVDs based on ATC codes from the PDR (online supplemental appendix C). Dispensed CVD medication prescriptions were included to increase the coverage of cases, given that CVDs are often diagnosed and followed-up in primary care services and may, therefore, not be captured in the NPR, which only includes specialist services. Medications used as secondary prevention of CVDs were not considered.

### Statistical analysis

We applied multivariable Cox proportional hazards regression analysis to assess the association between CVDs and candidate predictors, with follow-up time in days as underlying time scale.

To assess model discrimination (ie, the ability of the model to correctly distinguish between those with and without the outcome), Harrell’s c-index and the receiver operating characteristic (ROC) curve with the area under the curve (AUC) were used.^s32^ The c-index and AUC can vary between 0.5 and 1, with 1 being a perfect discrimination. To assess model calibration we used the integrated Brier score, which measures the average discrepancies between observed outcome status and estimated predictive values, at all times during the follow-up, with values between 0 and 1 (values closer to 0 indicate better calibration).^s33^ A bootstrapping method was used for internal validation of the derived model. Bootstrapping was applied by creating 200 bootstrap samples drawn from the total sample with replacement to provide performance estimates corrected for overfitting.^s34^

We presented sensitivity, specificity, positive predictive value (PPV) and negative predictive value (NPV) across two, previously established, high-risk thresholds of predicted probability set at 10% from a 5-year CVD risk prediction model^s35^ and 20% from a 10-year model in the general population.^s36^ These high-risk thresholds were selected as there were no available prediction models with shorter follow-up times for cardiovascular risk. To assess the added predictive ability of novel risk factors, we calculated the Net Reclassification Index (NRI), which summarises reclassification of participants when new predictors are added based on the two predefined thresholds. We also calculated two category-free/continuous measures, the category-free NRI^s37 s38^ and the Integrated Discrimination Improvement (IDI) index^s39^, which cover all possible thresholds of predicted probability. Positive values of the NRI and IDI index indicate improved performance.

We performed two post hoc analyses (not specified by the protocol) to test: (1) model performance in subgroups (ie, males/females, dispensed stimulant/non-stimulant medication, younger than 40/aged 40 and older)^6^ and (2) sensitivity, specificity, PPV, NPV and NRI for a wider range of cutoffs based on percentiles of predicted probabilities (online supplemental appendix J).

Data management was done using SAS V.9.4 (SAS Institute). R V.4.0.5 was used for data analysis, namely ‘survival’ and ‘rms’ package to generate the model and calculate C-index corrected for overfitting, ‘risksetROC’ package to create ROC curve, ‘ipred’ package to calculate the integrated Brier score, ‘rms’ package to create calibration plots, ‘survIDINRI’ package to calculate the NRI and IDI, and ‘mice’ package for multiple imputations.

### Missing data

In case that a variable had more than 30% of data missing, it would be deleted. We applied multiple imputations with 20 imputations for variables with less than 30% of data missing at random. We fit the analysis in each imputed data set using a regression model with all candidate predictors as explanatory variables, and combined parameter estimates across repeated analyses using Rubin’s rules.^s40 s41^ Multiple imputations were applied for variables educational attainment (missing data for 545 individuals, 2.2% of the cohort) and country of origin (missing data for four individuals, 0.02%).

## Findings

### Cohort description

Our cohort included 24 186 adults initiating ADHD pharmacological treatment. Baseline characteristics are presented in table 1 and online supplemental appendix E. By the end of the follow-up, 413 individuals had a CVD (1.7%), out of which 244 were male (59.2%).

**Table 1:** Descriptive information of the cohort

	N	%
Total study population	24 186	
Male	13 113	54.2
Female	11 073	45.8
Age at treatment start all: median (IQR)	33 (25–42)	
All CVDs	413	1.7
Male	244	1.9 (of males)
Female	169	1.5 (of females)
Age at CVD: median (IQR)	44 (33–52)	
Frequencies across diagnostic categories and medication prescriptions		
Ischaemic heart disease	49	0.2
Cerebrovascular disease	61	0.2
Venous thrombo-embolism	85	0.3
Heart failure	23	0.1
Tachyarrhythmias	112	0.5
CVD medication prescription	125	0.5

CVDs, cardiovascular diseases.

### Predictors of cardiovascular disorders

The model included eight traditional risk factors: age at treatment start, sex, hypertension, diabetes mellitus, obesity, hyperlipidaemia, tobacco use disorder and family history of CVD, and 4 out of 16 candidate novel risk factors: substance use disorder other than alcohol and tobacco, mood stabilisers, antipsychotics and substance use disorder medication (table 2). The traditional risk factor with the strongest association with CVD was diabetes mellitus (types 1 and 2) (HR 1.95, 95% CI 1.34 to 2.85, p<0.001). Among novel risk factors, substance use disorders (other than alcohol and tobacco) showed the strongest association with CVDs (HR 1.55, 95% CI 1.25 to 1.92, p<0.001).

**Table 2:** Associations between CVDs and predictors included in the model presented as HRs with 95% CIs

	Predictor	HR, 95% CI
	Traditional risk factors	
1	Age at treatment start	1.06 (1.05 to 1.06)*
2	Sex	0.80 (0.66 to 0.98)†
3	Hypertension	1.66 (1.31 to 2.10)*
4	Type 1 and type 2 diabetes mellitus	1.95 (1.34 to 2.85)*
5	Obesity	1.20 (0.78 to 1.82)
6	Hyperlipidaemia	0.96 (0.64 to 1.44)
7	Tobacco use disorder	1.84 (1.05 to 3.21)†
8	Family history of CVD	1.28 (1.02 to 1.61)†
	Novel risk factors	
9	Substances use disorder (other than tobacco and alcohol)	1.55 (1.25 to 1.92)*
10	Mood stabilisers	1.28 (1.01 to 1.66)†
11	Antipsychotics	1.32 (1.06 to 1.65)†
12	Substance use disorder medication	1.25 (0.97 to 1.61)‡

Note: Significance codes: *0.001; †0.05; ‡0.1.

CVD, Cardiovascular disease.

### Model performance measures and incremental value of novel risk factors

**Figure 1:**
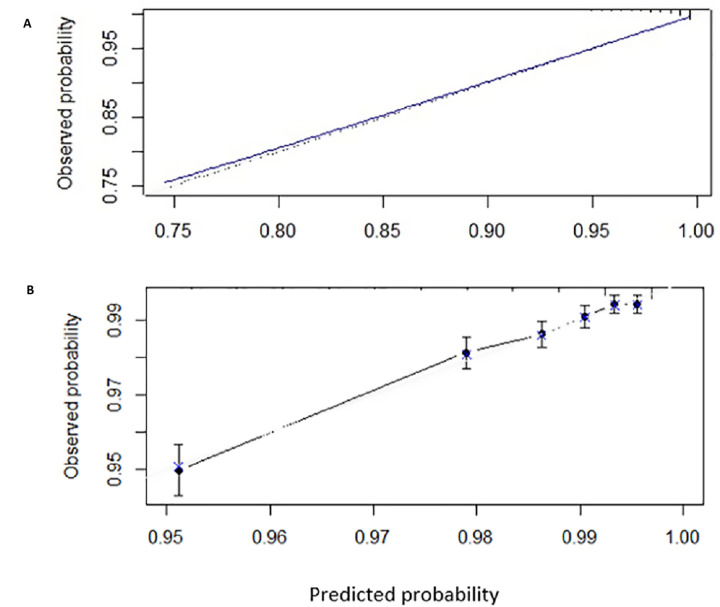
Calibration plots comparing predicted and observed probability of a 2-year cardiovascular disease-free survival/status (98.3% of the cohort) with grey line representing ideal calibration (A) each dot in black represents the observed proportion and each dot in blue represents the predicted proportion corrected for optimism. (B) Individuals are grouped according to percentiles of predicted probabilities of a disease-free survival, the vertical bars are 95% CIs (optimism corrected).

The model with traditional and novel CVD risk factors showed acceptable overall discrimination (C-index corrected for overfitting=0.72, 95% CI 0.70 to 0.74) (see online supplemental appendix F, for ROC curve) and calibration (integrated Brier score corrected for overfitting=0.008, see figure 1 for the calibration plot). Post hoc analyses showed that the model performance was mostly consistent across different subgroups (online supplemental appendix J).

Measures of model discrimination were lower in the model with only traditional risk factors (C index/AUC=0.70), while measures of calibration remained similar (integrated Brier score=0.008). The IDI index, with a value of 0.003 (95% CI 0.001 to 0.007), p<0.001, and a category-free or continuous NRI of 0.16 (95% CI 0.11 to 0.22), p<0.001, showed a significant improvement after adding novel risk factors.

Sensitivity, specificity, PPV and NPV are presented in table 3 for the model with traditional risk factors only, and the model including both traditional and novel CVD risk factors across high-risk thresholds set at 10%^s35 s42^ and 20%^s36^ of predicted probability. The NRI for the prespecified high-risk thresholds did not show a significant improvement when comparing the model with traditional risk factors only, with the model with traditional and novel CVD risk factors (table 3). These results remained consistent in a wider range of cutoffs of predicted probabilities (online supplemental appendix J).

**Table 3:** Sensitivity, specificity, positive predictive value (PPV), negative predictive value (NPV) and Net Reclassification Index (NRI) for prespecified high-risk thresholds based on actual cut-off points with corresponding risk scores percentiles for a model containing traditional risk factors only (model 1), and a model containing both traditional and novel risk factors (model 2)

Cut-off	Percentile	Model	Sensitivity	Specificity	PPV	NPV	NRI, 95% CI
20%	90^th^	1	0.34	0.91	0.10	0.98	0.01 (−0.02 to 0.02)
	89^th^	2	0.38	0.90	0.10	0.98	
10%	65^th^	1	0.64	0.66	0.05	0.98	0.002 (−0.023 to 0.025)
	66^th^	2	0.66	0.66	0.06	0.98	

Note: Confusion matrices are presented in online supplemental appendix G.

## Discussion

This study aimed to develop and internally validate a 2-year risk prediction model of CVD in a population of 24 186 adults initiating ADHD pharmacological treatment in Sweden using national health registries. By the end of the 2-year follow-up, 413 adults presented with CVDs (1.7%). Previous risk prediction models of CVD have not considered risk factors that may be relevant for individuals with ADHD, thus the current risk prediction model included novel risk factors associated with both ADHD and cardiovascular risk, in addition to traditional CVD risk factors. Our model showed that the inclusion of the novel CVD risk factors improved the accuracy of predicting cardiovascular risk in individuals initiating ADHD pharmacological treatment.

Our model including traditional and novel CVD risk factors yielded a C-index/AUC of 0.72, which is considered acceptable in CVD risk prediction models^s42^ and also comparable to other commonly used CVD prediction models (eg, Framingham risk score, QRISK, ASSIGN) with the AUC ranging from 0.68 to 0.88 across both sexes.^6^ Despite these findings, it is important to highlight that the present prediction model is not intended to be used in clinical practice, as studies of external validation and model updating are needed.^s43^ More research is needed on this topic as we were unable to identify other comparable studies on cardiovascular risk prediction conducted in the context of individuals with psychiatric disorders.

Several measures were used to assess the incremental value of adding new predictors. First, the NRI based on the two predefined high-risk threshold of 10%^s35^ and 20%^s36^ did not show significant improvements. On the other hand, category-free measures of the incremental value (ie, NRI and IDI index), which are considered as more objective measures of increment and less dependent on the outcome prevalence,^s44^ suggest a statistically significant improvement after adding novel risk factors to the model.

The PPVs of the two predefined thresholds were very low in this study. This was expected, given that the PPV depends on a population prevalence/incidence of a disorder and the incidence rate of CVDs in this study was low. This is probably due to the younger age of the study population (median age: 33 years) and the short follow-up time of 2 years, compared with previous cardiovascular risk prediction models that have been developed in older-aged populations (median age: over 50 years) and often using a longer follow-up time of at least 5 years.^6^

The current model included eight traditional CVD risk factors: age at treatment start, sex, diabetes mellitus, hypertension, obesity, hyperlipidaemia and tobacco use disorder, and family history of CVDs; and four novel risk factors: substance use disorders (other than tobacco and alcohol), mood stabilisers, antipsychotics and substance use disorder medication. Our findings regarding the non-traditional CVD risk factors are in line with previous studies.^s45-s52^ A systematic review and meta-analysis have showed that opium use is a significant risk factor for coronary artery diseases and ejection fraction in patients who had undergone heart surgery.^s45^ Similarly, medication used for treatment of opioid (ie, methadone) and alcohol dependence (ie, disulfiram), have been associated with cardiovascular complications, such as cardiac arrhythmia, changes in blood pressure and transient ischaemic changes.^s46-s48^ Additionally, previous research indicates that treatments with antipsychotic medication and mood stabilisers are associated with an increased risk for myocardial infarction,^s49^ stroke, sudden cardiac death and myocarditis.^s50^ Use of additional psychotropic medication in patients treated with ADHD medication is very common due to high psychiatric comorbidity.^s51^ Previous studies have shown that psychiatric polypharmacy (ie, treatment of psychiatric disorders with two or more medications) can lead to poor medication adherence and a greater risk of side effects.^s52^

### Clinical implications

Considering the low PPV and low sensitivity when using high-risk thresholds, and the significant improvement of the model after adding novel risk factors when using category-free measures, our model may provide an improved prediction when used as a continuous probability score. Previously used thresholds for identifying individuals at high-risk for CVD may need adjustments when applied to younger and middle-aged populations who are followed for shorter periods of time. Additionally, it will be necessary to consider clinicians’ views on appropriate thresholds.

Our findings validate the relevance of traditional CVD risk factors (ie, hypertension, obesity, diabetes mellitus), their increased monitoring, and preventative strategies for individuals at risk on the initiation of ADHD pharmacological treatment. Additionally, our findings regarding substance use disorders and psychotropic medication (ie, mood stabilisers, antipsychotics and substance use disorder medication) may suggest that these factors should be considered on top of the available recommendations from consensus statements^16^ and treatment guidelines^15^ in relation to traditional CVD risk factors. That is, clinicians and researchers need to explore if patients initiating ADHD pharmacological treatment who are at higher risk of developing CVDs should be more closely monitored in relation to their use of other psychotropic medication and substance use disorders.

### Strengths and weaknesses of this study

The current prediction model used data from a large, population-based sample in Sweden. The model shows good discrimination, and information on the included predictors can be easily collected during a clinical interview and medical history report. All predictors (with the exemption to age at treatment start) are binary and only demand that patients recollect whether they have acquired a certain diagnosis or medication prescription in the past. With regard to included medication as predictors, examples of medication and common indications should be provided to an individual with ADHD.

Certain limitations of this study should be considered. First, we used a backward stepwise approach in the process of selecting novel CVD risk factors, while keeping traditional risk factors fixed in the model. This approach allowed us to retain traditional CVD risk factors and test novel CVD risk factors, while keeping the final model simple and easily applicable in clinical practice. Nevertheless, the backward stepwise procedure may lead to overfitting,^s53^ which was partially addressed by using bootstrapping to correct the performance estimates. Thus, future external validation studies are warranted.

Second, some of the considered risk factors, such as tobacco use disorder and obesity, may only include more severe cases in our populations since the recorded data are based on diagnosed individuals within specialist care. Future validation studies of the model using self-reported or routinely collected information on smoking, weight and height could provide a better coverage of all affected individuals. Third, validation studies using more detailed assessments of other traditional and novel CVD risk factors, such as blood pressure, lipid values, use of other medication, current substance abuse, as well as physical activity, nutrition and other lifestyle factors^s54^ are needed, as this study was limited to data on recorded clinically diagnosed conditions.

Fourth, the follow-up time in traditional cardiovascular risk prediction models is commonly 5 or 10 years.^6^ The present model followed the included individuals for only 2 years from the start of ADHD treatment initiation. As a consequence of the short follow-up time and relatively young age of the population, the 2-year incidence of CVDs in the current study was 1.7%. The median age-standardised prevalence rates in the general population across Europe in 2017 for all CVDs were 7.1% in men and 6% in women.^s55^ Among US adults (aged 18–64), receiving ADHD pharmacological treatment, the prevalence of any CVD was estimated as 5.5%.^s56^ Future prediction modelling studies in the context of ADHD need to cover older individuals and a longer follow-up time. Future work will also need to consider the modelling of risk factors in low base rate outcomes^s57^ and using more prevalent outcomes (eg, elevation of CVD blood markers) could be considered.

## Conclusion

This study suggests that inclusion of the novel risk factors may improve the prediction of CVDs in individuals with ADHD compared with a model with traditional CVD predictors only. Nevertheless, future external validation studies and studies assessing clinical impact of the model are warranted.

## Data Availability

Data may be obtained from a third party and are not publicly available. The Public Access to Information and Secrecy Act in Sweden prohibits individual-level data to be publicly available. Researchers who are interested in replicating this study can apply for individual level data at Statistics Sweden: www.scb.se/en/services/guidance-for-researchers-and-universities/
