# Supplementary material for: Risk prediction model for cardiovascular diseases in adults initiating pharmacological treatment for attention-deficit/hyperactivity disorder
Source: Evid Based Ment Health. 2022 Sep 5;25(4):185–90. doi: 10.1136/ebmental-2022-300492 (PMC9685689; doi:10.1136/ebmental-2022-300492)
Supplement: Supplementary data [file ebmental-2022-300492supp001.pdf]

**Supplementary material**

**A risk prediction model for cardiovascular diseases in adults initiating pharmacological treatment for Attention-deficit/Hyperactivity Disorder**

Maja Dobrosavljevic, Seena Fazel, Ebba Du Rietz, Lin Li, Le Zhang, Zheng Chang, Tomas Jernberg, Stephen Faraone, Johan Jendle, Qi Chen, Isabell Brikell, Henrik Larsson

**Content:**

**Appendix A:** List of candidate predictors *Page 2*

**Appendix B:** International Classification of Diseases (ICD)-8/9/10 codes and the Anatomical Therapeutic Chemical (ATC) Classification System codes for candidate predictors *Page 3*

**Appendix C:** International Classification of Diseases (ICD)-10 codes and the Anatomical Therapeutic Chemical (ATC) Classification System codes for outcomes *Page 5*

**Appendix D:** Process of predictors selection *Page 7*

**Appendix E:** Baseline characteristics of the population with regards to predictors *Page 8*

**Appendix F:** Cardiovascular risk prediction model discrimination shown by the receiver-operating characteristics curve *Page 10*

**Appendix G:** Confusion matrices *Page 11*

**Appendix H:** Study protocol *Page 12*

**Appendix I**: Excess references *Page 18*

**Appendix J.** Post hoc analyses (analyses not prespecified by study protocol) *Page 20*



Dobrosavljevic M, *et al. Evid Based Ment Health* 2022; 25:185–190. doi: 10.1136/ebmental-2022-300492

**Appendix A: List of candidate predictors**

| Traditional risk factors | | | Non-traditional risk factors | | |
|---|---|---|---|---|---|
| | Variable | Data source and type | | Variable | Data source and type |
| 1 | Age | Total population register, continuous (years of age at treatment initiation) | 9 | Anxiety | National patient register, Binary, ICD-8,9,10 codes |
| 2 | Sex | Total population register, binary (male/female) | 10 | Depression | |
| 3 | Hypertension | National patient register, Binary, ICD-8,9,10 codes, and Prescribed drug register, Binary, ATC codes | 11 | Bipolar disorder | |
| 4 | Type 1 and Type 2 Diabetes mellitus | | 12 | Schizophrenia-spectrum disorders | |
| | | | 13 | Sleep disorders | |
| 5 | Hyperlipidemia | | 14 | Alcohol use disorder | |
| | | | 15 | Substance use disorder other than tobacco and alcohol | |
| 6 | Obesity | National patient register, Binary, ICD-8,9,10 codes | 16 | Educational attainment | LISA, Binary, High (0): secondary, post-secondary/university education; and low (1): in/complete elementary school |
| 7 | Tobacco use disorder | | 17 | Country of origin | Total population register, Non-Swedish/Swedish born |
| 8 | Family history of CVD (first degree relatives before age 60) | | 18 | Other psychotropic medication* | Prescribed drug register, Binary, ATC codes |

Note: LISA - Longitudinal integration database for health insurance and labour market studies register; ICD – International Classification of Diseases; ATC - Anatomical Therapeutic Classification.
*Each medication from this group will be considered as a separate predictor

**Appendix B: International Classification of Diseases (ICD)-8/9/10 codes and the Anatomical Therapeutic Chemical (ATC) Classification System codes for candidate predictors**

| | | ICD-8 | ICD-9 | ICD-10 | ATC |
|---|---|---|---|---|---|
| Hypertension | | 400-404 | 401-405 | I10-I13, I15 | C02; C03; C07; C08; C09 |
| Diabetes mellitus | | 250 | 250 | E10-E14 | A10A, A10B |
| Obesity | | 277.99 | 278A, 278B | E65-E66 | - |
| Hyperlipidemia | | 279 | 272 | E78 | C10 |
| Tobacco use disorder | | – | 305B | F17, T65.2, Z71.6, Z72.0 | - |
| Personal and family history of CVDs* | | 410-414, 430-438, 451.00, 451.98, 451.99, 450, 427.00, , 427.92, 427.90, 427.91 | 410-414, 430-438, 451, 415B, 428, 427D, 427A, 427B, 427E, 427F | I20-I25, I60-I69, I80, I26, I50, I48, I47.1, I47.0, I47.2, I49.0, I49.8, I46 | - |
| Anxiety | | 300 (except 300.4) | 300 (except 300E) | F40, F41, F42, F44, F45, F48 | |
| Depression | | 296.0, 300.4 | 296B, 300E, 311 | F32, F33, F34.1, F34.8, F34.9, F38.1 | |
| Bipolar disorder | | 296.10, 296.30, 296.80 | 296A/C/D/E/W | F30–F31 | |
| Schizophrenia | | 295.00–295.40, 295.60, 295.80–295.90 | 295A–295E, 295G, 295W, 295X | F20 | |
| Substance use disorder | All (except smoking) | 303, 304 | 303, 304, 305 (except 305B) | F10 – F19 (except F17) | |
| | Alcohol | 303 | 303, 305A | F10 | |
| | Other substances | 304 | 304, 305X | F11-F19, except F17 | |

| | | | | |
|---|---|---|---|---|
| Sleep disorders | 347, 780.60 | 347, 780F | G47.0/1/2/3/4/8/9, F51 | |
| | ATC | | | |
| Anxiolytics | N05B | | | |
| Hypnotics and sedatives | N05C | | | |
| Antiepileptics | N03A, except N03AG01, N03AX09, N03AF01, N03AF02 | | | |
| Antidepressants | N06A | | | |
| Mood stabilizers | N03AG01, N03AX09, N03AF01, N03AF02, N05AN01 | | | |
| Antipsychotics | N05A, except N05AN01 | | | |
| Drugs used for addictive disorders (alcohol and opioid dependance) | N07BB01, N07BB03, N07BB04, N02AE01, N07BC01, N07BC02, N07BC51 | | | |

*Definition of personal history of CVDs includes medication for CVD (see table Appendix C) in addition to ICD 8/9/10 codes

**Appendix C: International Classification of Diseases (ICD)-10 codes and the Anatomical Therapeutic Chemical (ATC) Classification System codes for outcomes**

| | ICD-10 | ATC |
|---|---|---|
| **Ischemic heart disease** | | C01D |
| Acute myocardial infarction | I21-I23 (primary) | |
| Acute myocardial infarction (also including secondary, type-2 myocardial infarction) | I21-I23 (primary or secondary) | |
| Acute coronary syndrome | I21-I23 or I20.0 | |
| Any ischemic heart disease (Chronic ischemic heart disease) | I20-I25 (primary or secondary) | |
| **Cerebrovascular disease** | | — |
| Subarachnoidal bleeding | I60 (primary or secondary) | |
| Hemorrhagic stroke | I61-I62 (primary or secondary) | |
| Ischemic stroke | I63-I64 (primary or secondary) | |
| Other cerebrovascular disease | I65-I69 (primary or secondary) | |
| **Venous thrombo-embolism** | | - |
| Deep vein thrombosis | I80 (primary or secondary) | |
| Pulmonary emboli | I26 (primary or secondary) | |

| | | |
|---|---|---|
| **Heart failure** | | C01A |
| Heart failure | I50 (primary diagnosis) | |
| Heart failure | I50 (primary and secondary) | |
| **Takyarrhythmias** | | |
| Atrial fibrillation/fludder | I48 (primary or secondary) | C01A |
| Supraventricular tachycardia | I47.1 (primary or secondary) | C01B |
| Ventricular tachycardia | I47.0, I47.2, I49.0, I49.8 (primary or secondary) | |
| Cardiac arrest | I46 (primary or secondary) | **-** |

*Uncommonly used substances in cardiology

**Appendix D: Process of predictors selection**

Variables were excluded consecutively based on their p-values: a variable with the highest p-value was excluded in each step of the analysis until all remaining predictors had p-vales lower than 0.1.

| Step. | Excluded predictor | estimate | std.error | statistic | df | p.value |
|---|---|---|---|---|---|---|
| 1. | Hypnotics and sedatives | -0.00 | 0.12 | -0.01 | 387.98 | 0.99 |
| 2. | Anxiety | 0.03 | 0.11 | 0.27 | 388.98 | 0.78 |
| 3. | Schizophrenia | -0.10 | 0.32 | -0.32 | 389.98 | 0.74 |
| 4. | Country of origin | -0.07 | 0.17 | -0.42 | 390.98 | 0.67 |
| 5. | Depression | -0.06 | 0.11 | -0.53 | 391.98 | 0.60 |
| 6. | Alcohol use disorder | 0.08 | 0.13 | 0.62 | 392.98 | 0.54 |
| 7. | Sleep disorders | -0.12 | 0.18 | -0.64 | 393.98 | 0.52 |
| 8. | Bipolar disorder | -0.16 | 0.18 | -0.92 | 394.97 | 0.36 |
| 9. | Educational attainment | 0.14 | 0.11 | 1.29 | 393.01 | 0.20 |
| 10. | Antiepileptics | 0.15 | 0.12 | 1.23 | 396.97 | 0.22 |
| 11. | Antidepressants | -0.18 | 0.12 | -1.51 | 397.97 | 0.13 |
| 12. | Anxiolytics | 0.15 | 0.11 | 1.41 | 398.97 | 0.16 |

**Appendix E: Baseline characteristics of the population with regards to predictors**

| | Predictor | Total N=24,186 | Male N=13,113 | Female N=11,073 | Individuals with CVD N=413 |
|---|---|---|---|---|---|
| 1 | Age at treatment start: median (IQR) | 33 (25-42) | 32 (25-42) | 33 (25-42) | 43 (32-51) |
| | | N (%) | | | |
| 2 | Sex (female) | 11,073 (45.78%) | - | - | 169 (40.92) |
| 3 | Hypertension | 2956 (12.22) | 1355 (10.33) | 1601 (14.46) | 119 (28.81) |
| 4 | Diabetes mellitus (type 1 and 2) | 645 (2.67) | 362 (2.76) | 283 (2.56) | 37 (8.96) |
| 5 | Obesity | 901 (3.73) | 278 (2.12) | 623 (5.63) | 25 (6.05) |
| 6 | Hyperlipidaemia | 718 (2.97) | 411 (3.13) | 307 (2.77) | 33 (7.99) |
| 7 | Tobacco use disorder | 309 (1.28) | 132 (1.01) | 177 (1.60) | 13 (3.15) |
| 8 | Family history of CVD (before age 60) | 4915 (20.32) | 2637 (20.11) | 2278 (20.57) | 98 (23.73) |
| 9 | Low educational attainment* | 8538 (36.11) | 5054 (39.55) | 3484 (32.08) | 140 (34.15) |
| 10 | Born in a country other than Sweden* | 2332 (9.64) | 1321 (10.07) | 1011 (9.13) | 38 (9.20) |
| 11 | Anxiety | 8803 (36.40) | 4031 (30.74) | 4772 (43.10) | 170 (41.16) |
| 12 | Depression | 8478 (35.05) | 3936 (30.01) | 4542 (41.02) | 158 (38.26) |
| 13 | Bipolar disorder | 2157 (8.92) | 824 (6.28) | 1333 (12.04) | 47 (11.38) |
| 14 | Schizophrenia | 343 (1.42) | 242 (1.85) | 101 (0.91) | 11 (2.66) |
| 15 | Alcohol use disorder | 4769 (19.72) | 2961 (22.58) | 1808 (16.33) | 128 (30.99) |
| 16 | Other substances use disorder | 6031 (24.93) | 3861 (29.44) | 2179 (19.60) | 162 (39.23) |
| 17 | Sleep disorders | 1620 (6.70) | 928 (7.08) | 692 (6.25) | 33 (7.99) |
| 18 | Anxiolytics | 12,113 (50.08) | 5743 (43.79) | 6370 (57.53) | 252 (61.02) |
| 19 | Hypnotics and sedatives | 12,301 (50.86) | 6125 (46.71) | 6176 (55.78) | 252 (61.02) |
| 20 | Antiepileptics | 3627 (15.00) | 1804 (13.76) | 1823 (16.46) | 101 (24.46) |
| 21 | Antidepressants | 16,121 (66.65) | 7733 (58.97) | 8389 (75.76) | 289 (69.98) |
| 22 | Mood stabilizers | 3864 (15.98) | 1792 (13.66) | 2072 (18.71) | 100 (24.21) |



Dobrosavljevic M*, et al. Evid Based Ment Health* 2022; 25:185–190. doi: 10.1136/ebmental-2022-300492

| | | | | | |
|---|---|---|---|---|---|
| 23 | Antipsychotics | 5609 (23.19) | 3010 (22.95) | 2599 (23.47) | 141 (34.14) |
| 24 | Drugs used for addictive disorders (alcohol and opioid dependance) | 2778 (11.49) | 1816 (13.85) | 962 (8.69) | 88 (21.31) |

*Missing data: Education category, N=545; Country of origin, N=4. Multiple imputation for missing data with 20 imputations were used.

**Appendix F: Cardiovascular risk prediction model discrimination shown by the receiver-operating characteristics (ROC) curve, with the false positive rate (1-specificity) on the x-axis and the true positive rate (sensitivity) on the y-axis.**



Dobrosavljevic M, *et al. Evid Based Ment Health* 2022; 25:185–190. doi: 10.1136/ebmental-2022-300492

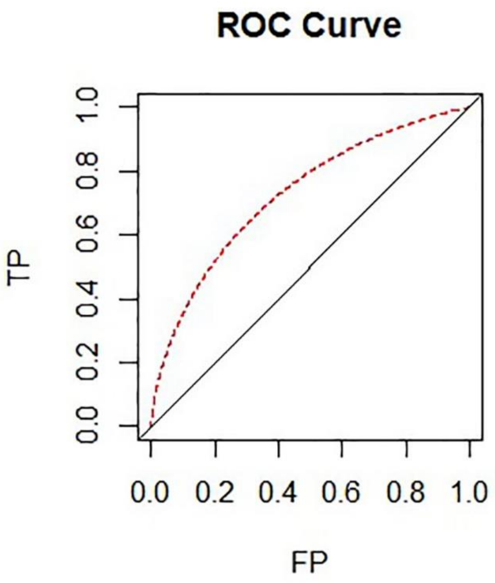


Note: TP-true positive, FP-false positive; The dotted line in red represents the ROC curve achieved by the model with the area under the curve (AUC)=0.73 (95% CI 0.71, 0.75), 0.72 (95% CI 0.70, 0.74) corrected for optimism; The solid line in black represents the ROC curves when AUC equals 50%.

**Appendix G: Confusion matrices for two prespecified thresholds and Model 1 (traditional CVD risk factors only) and Model 2 (with added non-traditional CVD risk factors) based on 200 bootstrap samples**

| **Threshold** | **Model 1** | Reference | | **Model 2** | Reference | |
|---|---|---|---|---|---|---|
| | | Non-event | Event | | Non-event | Event |

| | | | | | | | | |
|---|---|---|---|---|---|---|---|---|
| **20%** | Predicted values | Non-event | TN: 11,749 | FN: 263 | Predicted values | Non-event | TN: 11,608 | FN: 249 |
| | | Event | FP: 1204 | TP: 160 | | Event | FP: 1345 | TP: 174 |
| **10%** | | | Non-event | Event | | | Non-event | Event |
| | Predicted values | Non-event | TN: 8567 | FN: 140 | Predicted values | Non-event | TN: 8630 | FN: 125 |
| | | Event | FP: 4386 | TP: 283 | | Event | FP: 4323 | TP: 298 |

Note: TN: true negative; FN: false negative; FP: false positive; TP: true positive

**Appendix H: Study protocol**

**Study summary**

*Design:* Retrospective cohort study

*Data sources:* Data on ICD-based diagnoses and ATC-based medication prescriptions will be merged from several Swedish national registers.

*Population and study period:* We will follow all individuals aged 18 and older who initiate pharmacological treatment for ADHD between January 1st, 2008 and December 31st, 2011, from the date of treatment initiation until a cardiovascular disorder (CVD) diagnosis, emigration, death, or the end of two years, which ever came first.

*Risk factors:* We will include relevant traditional risk factors (age, sex, history of hypertension, diabetes mellitus, obesity, hyperlipidaemia, tobacco use disorder, and family history of CVDs), psychiatric disorders (history of depression, anxiety, alcohol use disorder, substance use disorder other than alcohol and tobacco, bipolar disorder, schizophrenia), previous use of other nervous system acting drugs, educational attainment, and country of origin.

*Outcomes:* First diagnosis of CVD after ADHD treatment initiation in the period between January 1st, 2008 and December 31st, 2011, within a two-year period.

*Outputs:* The main aim of the current study is to create a two-year risk model that will estimate the probability of obtaining a diagnosis of CVDs, with appropriate measures of predictive accuracy, and to obtain a scoring system for the calculation of risk at the time of ADHD treatment initiation.

## Methods

### Data sources

Data from multiple Swedish national registers will be merged, including the National Patient Register (NPR), Cause of Death Register (CDR), Prescribed Drug Register (PDR), and Longitudinal integration database for health insurance and labour market studies register (LISA). All diagnoses in the NPR and CDR were classified according to the International Classification of Diseases (ICD) versions 7/8/9/10. The Prescribed Drug Register (PDR) covers data on all dispensed medication prescriptions since July 1st, 2005, until December 31st, 2013, using the Anatomical Therapeutic Classification (ATC) system, with a date of prescription and dosage. Individuals from our study population will be linked with their first-degree relatives (parents and full siblings) using the Multi-generation Register, and information on family history of CVDs will be extracted from the NPR.

### Population and study period

Our cohort will be consisted of individuals aged ≥ 18 and born between 1932 and 1990, who had started pharmacological treatment for ADHD (Zetterqvist et al., 2013) between January 1st, 2008 and December 31st, 2011, and without previous history of CVDs. The inclusion period starts on January 1st, 2008, to allow for a wash-out period of two years for previous ADHD medication prescription, and previous prescriptions for other medication considered in the model. The inclusion period ends with December 31st, 2011, to allow for a two-year follow up, since the last available date of access for the study authors in the registers is December 31st, 2013. Individuals from the cohort will be followed from the starting date of the treatment until they developed a diagnosis of CVD, emigrated from Sweden, died, or by the end of two years.

We will include individuals with dispensed medication prescriptions for the following: Amphetamine (ATC code N06BA01), Dexamphetamine (ATC code N06BA02), and Methylphenidate (ATC code

N06BA04), and non-stimulant medication: Atomoxetine (ATC code N06BA09). Medication prescriptions for ADHD will be acquired from derived data based on natural language processing models for free-text prescriptions from the PDR, including medication prescription between 2006 and 2013 (Zhang et al., 2021). Prescriptions that were returned to pharmacies by patients after dispensation will be removed from the analysis. Furthermore, prescriptions for indications other than ADHD (e.g., narcolepsy, multiple sclerosis, idiopathic hypersomnia, and myotonic dystrophy) will be excluded from the analysis.

**Candidate predictors**

Previous well established risk prediction models for coronary heart disease include traditional risk factors such as: hypertension/hypertensive therapy, body mass index (BMI), smoking, diabetes, total/LDL/HDL cholesterol (Wilson et al., 1998) and family history of CVDs (Tunstall-Pedoe et al., 1997). We will select predictors a priori, based on previously established risk prediction models and relevant literature (i.e., systematic reviews and meta-analyses when available) and expert opinion. We will consider relevant somatic disorders, psychiatric disorders, and low educational attainment, associated with an increased risk for CVDs (Hennekens et al., 2005; Cohen et al., 2015; Kubota et al., 2017), which are also potential outcomes of ADHD (Nigg, 2013; Kooij et al., 2019); and previous use of nervous system acting drugs other than ADHD medication, which are commonly used for treatment of ADHD comorbidities and which have been associated with an increased risk for CVDs (Correll et al., 2015). All relevant diagnoses, medication prescriptions and socio-demographic information will be acquired by the date of first ADHD medication prescription.

We will include traditional CVD risk factors which are available in Swedish health registers: age, sex, a diagnosis of hypertension, diabetes mellitus, hyperlipidaemia, obesity, and smoking and family history of CVDs before age 60.

Non-traditional risk factors that will be considered are variables with prior evidence of associations with CVDs and/or ADHD: psychiatric disorders (i.e., depression, anxiety, alcohol use disorder, substance use disorder other than alcohol and tobacco, schizophrenia, bipolar disorder), previous use of other central nervous system agents (anxiolytics, antidepressants, hypnotics and sedatives, antiepileptics, mood stabilizers, antipsychotics and drugs used for addictive disorders), and demographic variables: educational attainment and country of origin (Sweden and other than Sweden). Anxiolytics may be used as a cardiovascular protective agent (Grundy et al., 2004; Balon et al., 2018), although some studies report an increased risk for cardiovascular-related death associated with the use of anxiolytics, and hypnotics and sedatives, combined with analgesics (Merlo et al., 1996), and an increased risk for a CVD event, associated with a combined used of antidepressants and anxiolytics (Krantz et al., 2009).

The model will use a backwards stepwise procedure to determine whether to retain these predictors based on their p-values (variables with the highest p value from the group of non-traditional risk

factors will be sequentially rejected, until none of them remain with a p-value greater than 0.1) (Royston and Sauerbrei, 2008), while holding fixed traditional risk factors.

The final model must present face validity. Interactions between predictors will not be considered in the model.

**Outcomes**

We will include an incident diagnosis (i.e., primary or any secondary diagnosis) or treatment prescription of the following CVDs: ischemic heart disease, cerebrovascular disease, venous thrombo-embolism, heart failure and tachyarrhythmias, after ADHD treatment initiation and within a two-year period. Cases of CVDs will be defined based on ICD-10 diagnostic codes from the NPR and CDR (Appendix B). We will include CVD medication prescription based on ATC codes from the PDR (Appendix B), to increase the coverage of cases, given that CVD conditions are often diagnosed and followed-up in primary care services and are, therefore, not covered by the NPR, which only includes specialist services. Medications used as secondary prevention of CVDs will not be considered, but only those used specifically for treatment of included CVDs.

**Statistical analysis**

We will apply Cox proportional hazards regression analysis to assess the association between CVDs and candidate predictors. A bootstrapping method will be used for internal validation of the model with 200 bootstrap samples. To assess discrimination of the model, ROC curve and c-index will be used. To assess calibration of the model we will use the Brier score (Brier, 1950) and calibration plots (by assessing the proportion of predicted and observed events at different levels of predicted probability) (Harrell et al., 1982). We will present sensitivity, specificity, positive predictive value (PPV) and negative predictive value (NPV) across two high-risk thresholds of predicted probability set at 10% from a 5-year CVD risk prediction model (Polonsky et al., 2010) and 20% from a 10-year model (Grundy et al., 2004).

We will also compare the performance of the model that includes only traditional risk factors with the model which includes additional non-traditional risk factors by calculating the following measurers of incremental value: the Net Reclassification Index (NRI), which summarizes reclassification of participants when new predictors are added based on the two predefined thresholds of predicted probability; two category-free or continuous measures: the category-free NRI (Pencina et al., 2008; Steyerberg et al., 2012) and the Integrated Discrimination Improvement (IDI) index (Kerr et al., 2011), which cover all possible cut-offs or thresholds of predicted probability.

**Missing data**

If less than 30% of data is missing at random, we will use multiple imputation with 20 imputations using a regression model with all candidate predictors as explanatory variables and the outcome variable (White and Royston, 2009; Bartlett et al., 2015). Otherwise, the predictor will be excluded from the model. We will fit the analysis in each imputed data set and combined parameter estimates using Rubin's rule.



Dobrosavljevic M*, et al. Evid Based Ment Health* 2022; 25:185–190. doi: 10.1136/ebmental-2022-300492

**Presentation of findings and model generalizability**

The main output of the model will be a predictive probability of occurrence of CVD from the date of ADHD pharmacological treatment initiation, within a two-year time-window. All risk factors and their estimated coefficients will be examined to create a model with face validity, and which is easy to use in practice (most of the predictors are binary). Considered candidate predictors should be easily assessed in clinical setting (i.e., medical history, medical examination, psychiatric assessment, etc.).

**Changes in the protocol after study completion:** To increase the participant coverage for the following predictors: hypertension, hyperlipidaemia, and diabetes mellitus (type 1 and 2), we additionally used dispensed medications for these conditions to identify affected individuals (see Appendix B for ATC codes). We also limited family history of CVD to only those individuals whose first-degree relatives acquired a CVD diagnosis before age 60, as it has been done in traditional CVD prediction models (Tunstall-Pedoe et al., 1997; Hippisley-Cox et al., 2017).

References:

Balon R, Rafanelli C and Sonino N (2018) Benzodiazepines: A Valuable Tool in the Management of Cardiovascular Conditions. *Psychotherapy and Psychosomatics* 87(6): 327-330.
Bartlett JW, Seaman SR, White IR, et al. (2015) Multiple imputation of covariates by fully conditional specification: Accommodating the substantive model. *Stat Methods Med Res* 24(4): 462-487.
Brier GW (1950) Verification of forecasts expressed in terms of probability. *Monthly weather review* 78(1): 1-3.

Cohen BE, Edmondson D and Kronish IM (2015) State of the art review: depression, stress, anxiety, and cardiovascular disease. *American journal of hypertension* 28(11): 1295-1302.
Correll CU, Detraux J, De Lepeleire J, et al. (2015) Effects of antipsychotics, antidepressants and mood stabilizers on risk for physical diseases in people with schizophrenia, depression and bipolar disorder. *World Psychiatry* 14(2): 119-136.
Grundy SM, Cleeman JI, Merz CN, et al. (2004) Implications of recent clinical trials for the National Cholesterol Education Program Adult Treatment Panel III guidelines. *Circulation* 110(2): 227-239.
Harrell FE, Califf RM, Pryor DB, et al. (1982) Evaluating the yield of medical tests. *Jama* 247(18): 2543-2546.
Hennekens CH, Hennekens AR, Hollar D, et al. (2005) Schizophrenia and increased risks of cardiovascular disease. *American heart journal* 150(6): 1115-1121.
Hippisley-Cox J, Coupland C and Brindle P (2017) Development and validation of QRISK3 risk prediction algorithms to estimate future risk of cardiovascular disease: prospective cohort study. *Bmj* 357: j2099.
Kerr KF, McClelland RL, Brown ER, et al. (2011) Evaluating the incremental value of new biomarkers with integrated discrimination improvement. *American Journal of Epidemiology* 174(3): 364-374.
Kooij J, Bijlenga D, Salerno L, et al. (2019) Updated European Consensus Statement on diagnosis and treatment of adult ADHD. *European psychiatry* 56(1): 14-34.
Krantz DS, Whittaker KS, Francis JL, et al. (2009) Psychotropic medication use and risk of adverse cardiovascular events in women with suspected coronary artery disease: outcomes from the Women's Ischemia Syndrome Evaluation (WISE) study. *Heart* 95(23): 1901-1906.
Kubota Y, Heiss G, MacLehose RF, et al. (2017) Association of educational attainment with lifetime risk of cardiovascular disease: the Atherosclerosis Risk in Communities Study. *JAMA internal medicine* 177(8): 1165-1172.
Merlo J, Hedblad B, Ögren M, et al. (1996) Increased risk of ischaemic heart disease mortality in elderly men using anxiolytics-hypnotics and analgesics. *European Journal of Clinical Pharmacology* 49(4): 261-265.
Nigg JT (2013) Attention-deficit/hyperactivity disorder and adverse health outcomes. *Clinical psychology review* 33(2): 215-228.
Pencina MJ, D'Agostino Sr RB, D'Agostino Jr RB, et al. (2008) Evaluating the added predictive ability of a new marker: from area under the ROC curve to reclassification and beyond. *Statistics in medicine* 27(2): 157-172.
Polonsky TS, McClelland RL, Jorgensen NW, et al. (2010) Coronary artery calcium score and risk classification for coronary heart disease prediction. *Jama* 303(16): 1610-1616.
Royston P and Sauerbrei W (2008) *Multivariable model-building: a pragmatic approach to regression anaylsis based on fractional polynomials for modelling continuous variables.* John Wiley & Sons.
Steyerberg EW, Pencina MJ, Lingsma HF, et al. (2012) Assessing the incremental value of diagnostic and prognostic markers: a review and illustration. *European journal of clinical investigation* 42(2): 216-228.
Tunstall-Pedoe H, Woodward M, Tavendale R, et al. (1997) Comparison of the prediction by 27 different factors of coronary heart disease and death in men and women of the Scottish Heart Health Study: cohort study. *Bmj* 315(7110): 722-729.
White IR and Royston P (2009) Imputing missing covariate values for the Cox model. *Statistics in medicine* 28(15): 1982-1998.
Wilson PW, D'Agostino RB, Levy D, et al. (1998) Prediction of coronary heart disease using risk factor categories. *Circulation* 97(18): 1837-1847.
Zetterqvist J, Asherson P, Halldner L, et al. (2013) Stimulant and non-stimulant attention deficit/hyperactivity disorder drug use: total population study of trends and discontinuation patterns 2006–2009. *Acta Psychiatrica Scandinavica* 128(1): 70-77.

Zhang L, Lagerberg T, Chen Q, et al. (2021) Prediction of treatment dosage and duration from free-text prescriptions: an application to ADHD medications in the Swedish prescribed drug register. *Evid Based Ment Health*. Epub ahead of print 2021/04/03. DOI: 10.1136/ebmental-2020-300231.

**Appendix I: Excess references**

31. Hippisley-Cox J, Coupland C, Vinogradova Y, et al. Performance of the QRISK cardiovascular risk prediction algorithm in an independent UK sample of patients from general practice: a validation study. *Heart* 2008;94(1):34-39.
32. Heagerty PJ, Zheng Y. Survival model predictive accuracy and ROC curves. *Biometrics* 2005;61(1):92-105. doi: 10.1111/j.0006-341X.2005.030814.x [published Online First: 2005/03/02]
33. Brier GW. Verification of forecasts expressed in terms of probability. *Monthly weather review* 1950;78(1):1-3.

34. Harrell FE, Jr., Lee KL, Mark DB. Multivariable prognostic models: issues in developing models, evaluating assumptions and adequacy, and measuring and reducing errors. *Stat Med* 1996;15(4):361-87.
35. Polonsky TS, McClelland RL, Jorgensen NW, et al. Coronary artery calcium score and risk classification for coronary heart disease prediction. *Jama* 2010;303(16):1610-16.
36. Grundy SM, Cleeman JI, Merz CN, et al. Implications of recent clinical trials for the National Cholesterol Education Program Adult Treatment Panel III guidelines. *Circulation* 2004;110(2):227-39. doi: 10.1161/01.Cir.0000133317.49796.0e [published Online First: 2004/07/14]
37. Pencina MJ, D'Agostino Sr RB, D'Agostino Jr RB, et al. Evaluating the added predictive ability of a new marker: from area under the ROC curve to reclassification and beyond. *Statistics in medicine* 2008;27(2):157-72.
38. Steyerberg EW, Pencina MJ, Lingsma HF, et al. Assessing the incremental value of diagnostic and prognostic markers: a review and illustration. *European journal of clinical investigation* 2012;42(2):216-28.
39. Kerr KF, McClelland RL, Brown ER, et al. Evaluating the incremental value of new biomarkers with integrated discrimination improvement. *American journal of epidemiology* 2011;174(3):364-74.
40. White IR, Royston P. Imputing missing covariate values for the Cox model. *Statistics in medicine* 2009;28(15):1982-98.
41. Bartlett JW, Seaman SR, White IR, et al. Multiple imputation of covariates by fully conditional specification: Accommodating the substantive model. *Stat Methods Med Res* 2015;24(4):462-87. doi: 10.1177/0962280214521348 [published Online First: 2014/02/15]
42. Lloyd-Jones DM. Cardiovascular risk prediction: basic concepts, current status, and future directions. *Circulation* 2010;121(15):1768-77. doi: 10.1161/circulationaha.109.849166 [published Online First: 2010/04/21]
43. Larsson H. Causation and prediction in child and adolescent mental health research: Wiley Online Library, 2021:e12026.
44. Pencina MJ, D'Agostino RB, Sr., Steyerberg EW. Extensions of net reclassification improvement calculations to measure usefulness of new biomarkers. *Stat Med* 2011;30(1):11-21. doi: 10.1002/sim.4085 [published Online First: 2011/01/05]
45. Nakhaee S, Amirabadizadeh A, Qorbani M, et al. Opium use and cardiovascular diseases: a systematic review and meta-analysis. *Crit Rev Toxicol* 2020;50(3):201-12. doi: 10.1080/10408444.2020.1740972 [published Online First: 2020/04/02]
46. Behzadi M, Joukar S, Beik A. Opioids and Cardiac Arrhythmia: A Literature Review. *Medical Principles and Practice* 2018;27(5):401-14. doi: 10.1159/000492616
47. Alinejad S, Kazemi T, Zamani N, et al. A systematic review of the cardiotoxicity of methadone. *EXCLI J* 2015;14:577.
48. Kumaraswamy G, Pundarikaksha H, Ramaiah V, et al. A study of cardiovascular complications of disulfiram-ethanol reaction. *National Journal of Physiology, Pharmacy and Pharmacology* 2013;3(1):35-42.
49. Huang K-L, Fang C-J, Hsu C-C, et al. Myocardial infarction risk and antipsychotics use revisited: a meta-analysis of 10 observational studies. *Journal of Psychopharmacology* 2017;31(12):1544-55. doi: 10.1177/0269881117714047
50. Correll CU, Detraux J, De Lepeleire J, et al. Effects of antipsychotics, antidepressants and mood stabilizers on risk for physical diseases in people with schizophrenia, depression and bipolar disorder. *World Psychiatry* 2015;14(2):119-36. doi: https://doi.org/10.1002/wps.20204
51. Zhang L, Reif A, Du Rietz E, et al. Comedication and polypharmacy with ADHD medications in adults: a Swedish nationwide study. *Journal of Attention Disorders* 2021;25(11):1519-28.
52. Sarkar S. Psychiatric polypharmacy, etiology and potential consequences. *Current Psychopharmacology* 2017;6(1):12-26.

53. Smith G. Step away from stepwise. *Journal of Big Data* 2018;5(1):32. doi: 10.1186/s40537-018-0143-6
54. Zhang Y-B, Pan X-F, Chen J, et al. Combined lifestyle factors, all-cause mortality and cardiovascular disease: a systematic review and meta-analysis of prospective cohort studies. *J Epidemiol Community Health* 2021;75(1):92-99.
55. Townsend N, Kazakiewicz D, Lucy Wright F, et al. Epidemiology of cardiovascular disease in Europe. *Nature Reviews Cardiology* 2021 doi: 10.1038/s41569-021-00607-3
56. Fairman KA, Davis LE, Peckham AM, et al. Diagnoses of cardiovascular disease or substance addiction/abuse in US Adults treated for ADHD with stimulants or atomoxetine: is use consistent with product labeling? *Drugs-real world outcomes* 2018;5(1):69-79.
57. Fanshawe TR, Fazel S. The 'double whammy' of low prevalence in clinical risk prediction. *BMJ Evid Based Med* 2021 doi: 10.1136/bmjebm-2021-111683 [published Online First: 2021/08/15]

**Appendix J. Post hoc analyses (analyses not prespecified by study protocol)**

1) Performance of the derived model across different subgroups

| | N | CVD cases | C-index with 95% CI | Brier score |
|---|---|---|---|---|
| Sex | | | | |
| Male | 13,113 | 244 | 0.75 (0.72, 0.78) | 0.009 |
| Female | 11,073 | 169 | 0.71 (0.67, 0.75) | 0.007 |
| Medication | | | | |

| | | | | |
|---|---|---|---|---|
| Stimulant | 20,462 | 345 | 0.73 (0.70, 0.76) | 0.008 |
| Nonstimulant | 3724 | 68 | 0.74 (0.69, 0.79) | 0.009 |
| Age group | | | | |
| Younger than 40 | 16,655 | 169 | 0.68 (0.64, 0.72) | 0.005 |
| Aged 40 and older | 7531 | 244 | 0.70 (0.67, 0.73) | 0.015 |

Note. CI: confidence interval

2) Sensitivity, specificity, positive predictive value (PPV), negative predictive value (NPV) and net reclassification index (NRI) for a model containing traditional risk factors only (Model 1), and a derived model containing both traditional and novel risk factors (Model 2) across different percentile risk thresholds

| Percentile | Model | Cutoff | Sensitivity | Specificity | PPV | NPV | NRI, 95% CI |
|---|---|---|---|---|---|---|---|
| 95th | 1 | 27.0% | 0.20 | 0.95 | 0.12 | 0.97 | 0.004 (-0.005, 0.019) |
| | 2 | 28.6% | 0.21 | 0.95 | 0.13 | 0.97 | |
| 90th | 1 | 20.2% | 0.34 | 0.91 | 0.11 | 0.98 | 0.01 (-0.01, 0.02) |
| | 2 | 21.1% | 0.34 | 0.91 | 0.11 | 0.98 | |
| 80th | 1 | 14.3% | 0.48 | 0.81 | 0.07 | 0.98 | -0.003 (-0.019, 0.0189 |
| | 2 | 14.7% | 0.52 | 0.81 | 0.08 | 0.98 | |
| 50th | 1 | 7.1% | 0.75 | 0.51 | 0.05 | 0.98 | 0.001 (-0.025, 0.032) |
| | 2 | 7.4% | 0.78 | 0.51 | 0.05 | 0.99 | |

Note. CI: confidence interval



Dobrosavljevic M, *et al. Evid Based Ment Health* 2022; 25:185–190. doi: 10.1136/ebmental-2022-300492
